# A High Accuracy & Ultra-Low Power ECG-Derived Respiration Estimation Processor for Wearable Respiration Monitoring Sensor †

**DOI:** 10.3390/bios12080665

**Published:** 2022-08-22

**Authors:** Jiajing Fan, Siqi Yang, Jiahao Liu, Zhen Zhu, Jianbiao Xiao, Liang Chang, Shuisheng Lin, Jun Zhou

**Affiliations:** School of Information and Communication Engineering, University of Electronic Science and Technology of China, Chengdu 611731, China

**Keywords:** EDR, processor, QRS detection, wearable respiration monitoring sensor

## Abstract

The respiratory rate is widely used for evaluating a person’s health condition. Compared to other invasive and expensive methods, the ECG-derived respiration estimation is a more comfortable and affordable method to obtain the respiration rate. However, the existing ECG-derived respiration estimation methods suffer from low accuracy or high computational complexity. In this work, a high accuracy and ultra-low power ECG-derived respiration estimation processor has been proposed. Several techniques have been proposed to improve the accuracy and reduce the computational complexity (and thus power consumption), including QRS detection using refractory period refreshing and adaptive threshold EDR estimation. Implemented and fabricated using a 55 nm processing technology, the proposed processor achieves a low EDR estimation error of 0.73 on CEBS database and 1.2 on MIT-BIH Polysomnographic Database while demonstrating a record-low power consumption (354 nW) for the respiration monitoring, outperforming the existing designs. The proposed processor can be integrated in a wearable sensor for ultra-low power and high accuracy respiration monitoring.

## 1. Introduction

Respiratory rate is an important index for evaluating a person’s health condition or diagnosing diseases. For example, the respiratory rate can be used to determine whether the patient has acute deterioration [1,2]. In [3], it is proposed that doctors can use an excessive respiratory rate to predict cardiopulmonary arrest. The respiration rate is also used in clinical treatment to diagnose hypercapnia [4], pneumonia [5], and other diseases.

Although the respiratory rate is very important for monitoring the patient’s health condition, its recording is still one of the rarest among the physiological signals [6,7]. One of the main reasons is that the conventional methods used to monitor the respiration rate, such as impedance plethysmography, capnography, and flow thermography, all rely on invasive and expensive equipment, making it uncomfortable and costly for patients to use [8].

The electrocardiogram (ECG) is often used to monitor heart activity non-invasively. It can also be used to estimate the respiration rate. The ECG is mainly composed of P wave, QRS complex and T wave. The QRS complex consists of Q wave, R peak and S wave, where the R peak is the main component of QRS complex due to its large amplitude and energy. The respiration rate can be extracted from the ECG based on the dynamic variation of the amplitude of QRS complex, that is, ECG-derived respiration (EDR). Compared with the conventional respiration rate monitoring methods, the EDR method is more affordable and comfortable [9].

Current EDR estimation methods are mainly divided into frequency-domain-based methods [10,11] and time-domain-based methods [12,13]. Among them, frequency-domain-based methods often require complex computations, such as complex filtering and modulation [10] or wavelet transform [11], making them unsuitable for low power and low-cost wearable respiration monitoring sensors.

For time-domain based methods, the accuracy of respiration rate estimation is dependent on the accuracy of QRS detection. However, the accuracy of QRS detection suffers from a variety of noises such as muscle noise, baseline drift, and motion artifacts, causing false negatives (FN) or false positives (FP) [14,15,16]. To address these challenges and improve the accuracy, many QRS detection methods employ complex signal processing such as discrete wavelet transform (DWT) [17,18], Shannon energy [19] and convolutional neural network (CNN) [20,21]. However, this significantly increases the computational complexity, making them difficult to implement in hardware with low power and cost for wearable devices.

In this work, a high accuracy and ultra-low power EDR estimation processor has been proposed. Several techniques have been proposed to improve the accuracy with low computational complexity, including QRS detection using refractory period refreshing and adaptive threshold EDR estimation. The proposed EDR estimation processor has been implemented using a 55 nm processing technology for the evaluation of performance and power consumption.

The rest of paper is organized as follows. Section 2 reviews the existing EDR estimation methods and QRS detection methods. Section 3 presents the proposed refractory period refreshing technique and adaptive threshold technique for improving the accuracy of QRS detection and EDR estimation. Section 4 presents the implementation details of the proposed EDR processor. Section 5 discusses the experimental results, and Section 6 concludes the paper.

## 2. Existing Work

### 2.1. ECG-Derived Respiration Methods

Typically, spirometry, pneumography, plethysmography, or capnography can be used to measure the respiratory rate. However, the invasiveness of these methods makes them inconvenient for patients to use [22]. Additionally, these methods rely on expensive equipment that is mainly used in the ICU. Another method with lower cost for measuring respiration rate is the manual calculation of chest wall motion, which is usually used in general wards. However, the accuracy of this method is relatively low [23].

**Figure 1:**
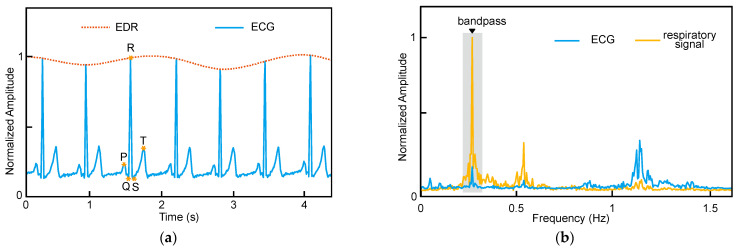
Illustration of EDR estimation principles. (**a**) Time-domain method. (**b**) Frequency-domain method.

To address these issues, ECG-derived respiration has been proposed, which can estimate the respiratory rate from the ECG signal. This method is non-invasive and low cost compared to the above-mentioned methods. The EDR estimation can be performed on the time or frequency-domain [10,12,13,22,24]. As shown in Figure 1a, in the time-domain, when a person is breathing, the electrodes used to measure the ECG will move in distance and direction with the ups and downs of the chest [25]. This will cause dynamic variation in the Q, R, S of the ECG signal as shown in Figure 1a. Therefore, the respiration rate can be estimated from these variations. Figure 1b shows EDR estimation in frequency-domain. A band-pass filter can be used to obtain the spectrum of ECG around 0.3 Hz (which well covers the frequency of respiration), and the respiration rate can be determined according to the peak of the spectrum. It can be seen from Figure 1b that the peak of the ECG spectrum after filtering well corresponds to the peak of the respiratory signal spectrum.

In the time-domain, the ECG amplitude and heart rate variability have been combined to estimate EDR through cubic spline interpolation and amplitude detection to improve the estimation accuracy [12,26]. In [13], the principal component analysis is utilized for EDR estimation based on the QRS detection and segmentation of ECG to further improve the accuracy. In the frequency-domain, the method of segment modulation has been proposed for the EDR estimation [10]. After removing high frequency noise, the continuous ECG is divided into individual heart beats using R peak detection. Then, the respiration information is removed through a segmented-beat modulation method, a robust template-based technique. After that, the difference of ECG before and after removing the respiratory information is obtained, and the respiration rate can be estimated. In [22], the empirical mode decomposition, Fourier transform and cubic spline interpolation are combined for the EDR estimation to improve the accuracy. Compared to the time-domain EDR methods, the frequency-domain EDR methods usually have higher complexity, which causes higher power consumption and hardware overhead.

### 2.2. QRS Detection Methods

For the time-domain EDR methods, the respiration rate is estimated from the dynamic variation of the QRS complex. The QRS detection method is aimed to detect the location of the QRS complex (mainly R peak) in the ECG signal. As the R peak has relatively high amplitude and energy, a threshold-based approach is commonly used for the R peak detection by comparing the amplitude of the ECG signal with a pre-defined threshold value. This method is relatively simple and easy to implement, but it is susceptible to noise and interference of other peaks. To address this issue, some improved threshold-based methods have been proposed. For example, a search-back method is introduced in [14]. If a QRS complex is not found during a long period defined by the average RR-interval (the average time interval between two adjacent R peaks), the peak that is lower than the threshold but with the highest amplitude since the last detected QRS complex is considered to be an R peak.

In the frequency domain, wavelet transform is an effective approach to analyze signals. A variety of wavelets, including Mexican hat wavelet [17], Mortlet’s wavelet [18], etc., have been used for QRS detection. In [17], the noise is attenuated with the Mexican hat wavelet and the Shannon energy of ECG signals is utilized to detect the R peak from QRS complex. In [18], a QRS detection method based on Mortlet’s wavelet with automatic scale selection corresponding to the maximum energy of the ECG signals is introduced.

With the rapid development of artificial intelligence (AI) technology, neural networks (NN) have been used in image recognition and object detection and have achieved high accuracy. In the meantime, NNs have been used for the QRS detection [20,21]. For example, Ref. [20] proposes a CNN-based QRS detector that learns fused features from multiple physiological signals. Being data-driven and learning features from arbitrary set of signals, this method is robust for different sets of signals. Another paper [21] proposes a method that that can directly detect QRS complex using Faster-RCNN by transforming the ECG signal into an image. The location is labeled as output after bounding-box regression.

Compared with the threshold-based methods, the frequency-domain methods and the NN-based methods achieve higher accuracy, but with significant computational complexity, which causes large power consumption and hardware overhead. This makes them unsuitable for power and cost constrained wearable devices.

## 3. Proposed EDR Estimation Method

### 3.1. Proposed QRS Detection Using Refractory Period Refreshing

**Figure 2:**
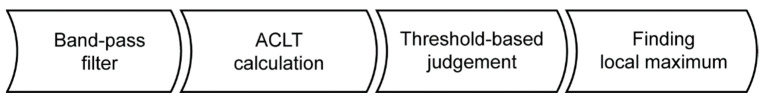
Block diagram of proposed QRS complex detection method.

The block diagram of QRS detection method is shown in Figure 2. In order to suppress noise and enhance the feature of QRS complex for QRS detection, the sampled ECG signal first goes through a band-pass filter built by cascading a low-pass filter and a high-pass filter as in [14]. The transfer function of low-pass filter with a cutoff frequency of about 11 Hz is shown in (1):

(1) $$H\left(z\right)=\frac{{\left(1-z^{-6}\right)}^{2}}{{\left(1-z^{-1}\right)}^{2}}$$

The transfer function of high-pass filter is shown in (2):

(2) $$H\left(z\right)=\frac{-1+32z^{-16}-32z^{-17}+z^{-32}}{1-z^{-1}}$$

where the cutoff frequency is about 5 Hz. The coefficients of the form $2^{n}$ where n is an integer allow the band-pass filter to be implemented with only shifters and adders without any multiplier. This reduces the hardware and power overhead.

To enhance the R peak for the QRS detection, a transformation named absolute curve length transform (ACLT) [27] is applied to the filtered signal, as shown in (3). Compared to other transformation methods, this method has relatively low computational complexity, as it does not involve multiplications and logarithms as in other transformation methods [19].

(3) $$L\left(\omega ,i\right)=\overset{i}{\underset{k=i-\omega }{\sum }}\left|C+\right|\Delta y_{k}||$$

where $\Delta y_{k}=y_{k}-y_{k-1}$ is the difference between two adjacent samples of filtered signal. C is a constant. The accumulation acts as a moving average filter, and ω is the length of the moving window. The purpose of the above operations is to suppress P and T wave and enhance the R peak.

This is followed by a thresholding operation. Firstly, to avoid false peaks, a refractory period mechanism is adopted that skips the peaks detected within the refractory period. Secondly, the threshold is adaptively adjusted according to the last couple of R peaks (e.g., eight R peaks) as the amplitude of the R peak may change over time. The adaptive threshold adjustment is shown in (4):

(4) $$Th_{adaptive}=\frac{\sum_{i-7}^{i}Rpeak_{i}}{M}$$

where M is a scaling factor used for adjusting the adaptive threshold. The M can be set to a value larger than 8 to make the threshold smaller than the average value of the eight R peaks to adjust the threshold as a scaling factor. In this work, we have investigated the records in the MIT-BIH Arrhythmia Database and found that the minimum RR-interval is greater than 90 samples (0.25 s), so we set the refractory period to 90. The refractory period mechanism can effectively reduce the FP. In the meanwhile, to reduce FN, we tried to reduce the threshold by increasing M in (4). However, we found that when we combine the refractory period mechanism and low threshold, the FP and FN are not the lowest, as shown in Table 1. Here, 3 methods are adopted: high threshold with refractory period (M1), low threshold without refractory period (M2) and low threshold with refractory period (M3). It can be seen from the table that the lowest FP appears in M1 and the lowest FN appears in M2. For M3, neither FP nor FN are the lowest.

**Figure 3:**
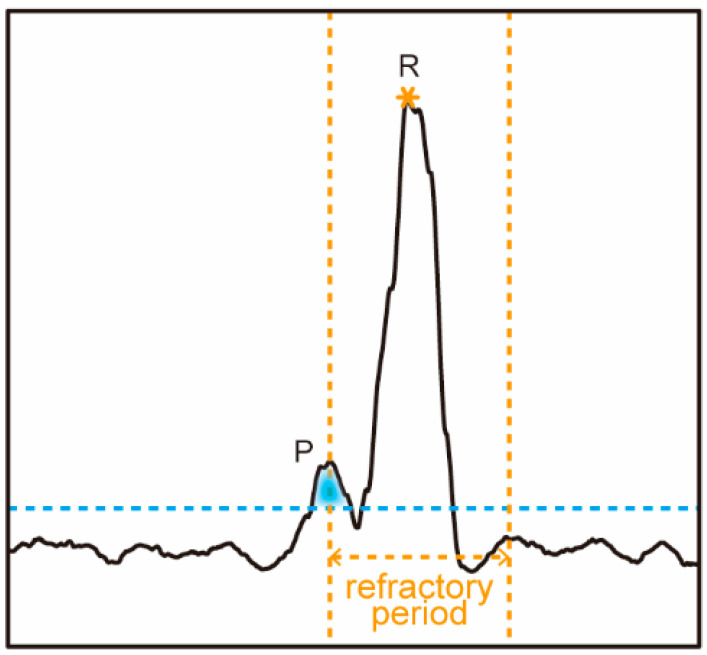
The R peak is blocked by P wave due to refractory period.

**Figure 4:**
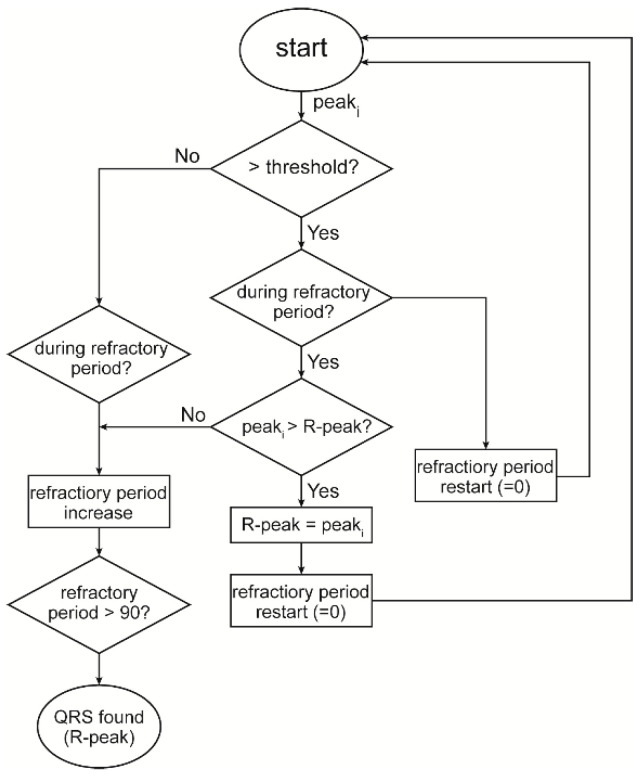
The flow diagram of threshold-based judgement with improved refractory period mechanism.

We investigated this issue and found the reason is that a low threshold will cause more false R peaks which blocks the true R peaks when the refractory period mechanism is adopted, as shown in Figure 3. It can be seen that when the threshold is low the P wave is also detected as peak, and this blocks the true R peak following it. To address this problem, we propose a refractory period refreshing mechanism of which the flow diagram is shown in Figure 4.

When a peak larger than the threshold is detected during the refractory period, instead of skipping it directly, its amplitude will be compared with the previous peak. If the current peak is larger than the previous peak, it is considered to be a potential R peak, and the previous peak is discarded. In the meanwhile, a new refractory period is started, and within this period if there is no peak larger than this peak, then this peak is considered as a true R peak. If not, then this peak is discarded and the larger peak is considered as a potential R peak that triggers another refractory period, and so forth. This effectively improves the FP and FN, as will be discussed in Section 5.

In addition, the locations of detected R peaks are inaccurate because the ECG signal has been pre-processed by band-pass filter and ACLT. Due to the delay of pre-processing, when a R-peak is detected, the corresponding R-peak in the original ECG waveform has passed. The total delay is:

(5) $$T_{total}=T_{bpf}+T_{ACLT}+T_{ref}$$

where $T_{bpf}$, $T_{ACLT}$, and $T_{ref}$ are the delay of band-pass filter, ACLT, refractory period, respectively. Hence, after the R peak is detected, we move to the original ECG waveform to perform a local maximum calculation. This is performed by finding the maximum in a small window (±16 samples) around the time point which is $T_{total}$ before the time point when the R peak is detected. The location of this maximum will be the real location of R peak. The location and amplitude of the S wave are found by detecting the minimum after the real location of R peak.

### 3.2. Proposed Adaptive Threshold Based EDR Estimation

For ECG-based EDR estimation, Fourier transform or counting methods are often used. For the EDR estimation using Fourier transform, the cubic spline interpolation is used to fill and smooth the obtained EDR signal. After that, the Fourier transform is used to extract the frequency spectrum of the signal, and the peak value is the respiratory frequency. Finally, this value is multiplied with 60 (seconds) to obtain the number of breaths per minute (bpm). The major issue of this method is that it involves the Fourier transform and cubic spline interpolation, resulting in large computational complexity.

Compared to the Fourier transform based method, the counting method has much lower complexity. In this method, first, the EDR signal is obtained from the ECG signal by tracking the variation of the QRS complexes as shown in Figure 1. Then, the EDR signal is segmented and a threshold is calculated in each segment. After that the respiration rate is estimated by counting the number of times that the EDR signal crosses the segmented threshold lines. There are two ways to calculate the threshold: using maximum value or average value in the segment, as shown in (6) and (7). $A_{max}$ is the highest value of the amplitude values extracted in a segment, and $A_{average}$ is the average value of the amplitude values extracted in this segment. The coefficients σ of these two equations are two configurable parameters, which are used to adjust the two threshold values. The (6) and (7) are used to explain the traditional threshold calculation method in the existing work. The traditional threshold calculation method is usually obtained by directly multiplying the maximum value or the average value by a coefficient [12].

(6) $$T_{max}=\sigma_{max}\times A_{max}$$

(7) $$T_{average}=\sigma_{average}\times A_{average}$$

**Figure 5:**
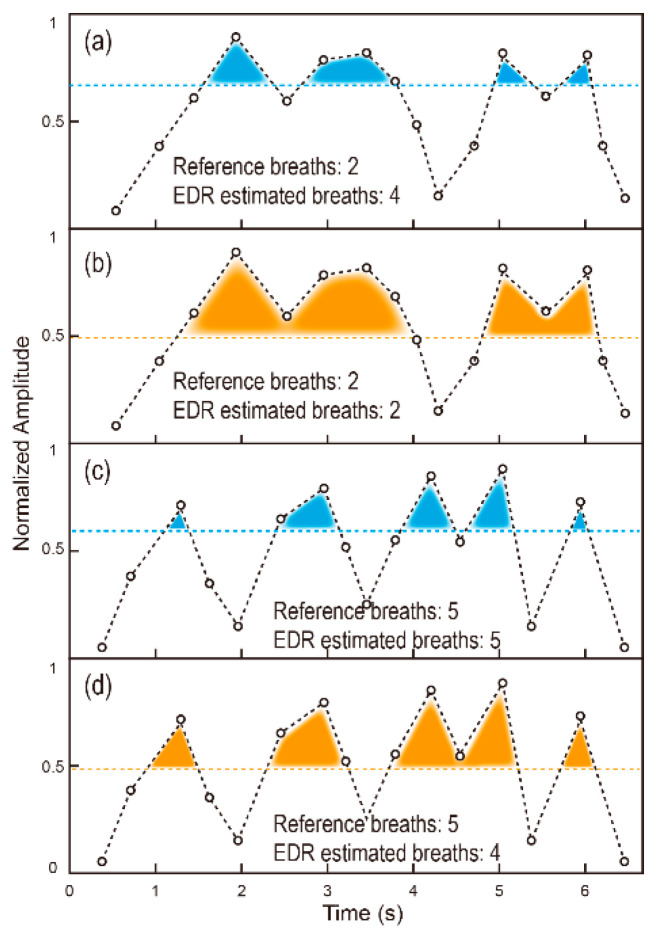
EDR estimation using the counting methods. (**a**,**c**) Threshold calculated using maximum value. (**b**,**d**) Threshold calculated using the average value.

Figure 5 shows the EDR estimation using the counting method, where Figure 5a,c are based on the maximum value and Figure 5b,d are based on the average value. In Figure 5a,b, the reference number of breaths is 2. In Figure 5c,d, the reference number of breaths is 5. As can be seen in Figure 5, both methods may end up with wrong number of breaths. When the respiration rate is high Figure 5c,d, the average value based method may lead to a relatively low threshold which results in less number of breaths. When the respiration rate is low Figure 5a,b, the maximum value-based method may lead to a relatively high threshold, which results in more number of breaths.

**Figure 6:**
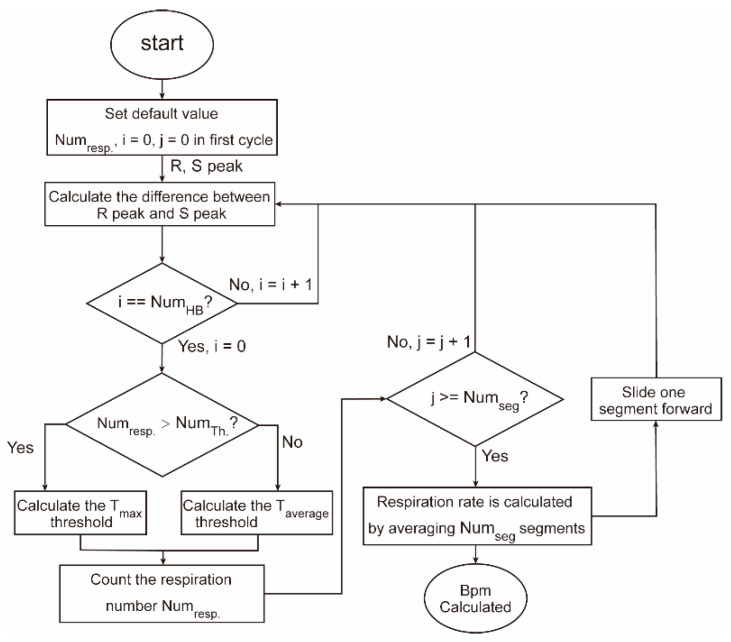
EDR estimation method flowchart.

In order to address this issue, we propose an adaptive threshold-based EDR estimation method, in which the threshold is adjusted according to the range of the respiration rate. The flowchart of the proposed EDR method is shown in Figure 6. The detailed method is described as follows:

Step 1: Calculate the difference between the amplitude of R peak and S peak from the QRS detection.

(8) $$A_{RS}=A_{R}-A_{S}$$

Step 2: Calculate the threshold values using both maximum value and average value in a pre-defined window containing $Num_{HB}$ R-S peaks.

(9) $$T_{max}=\frac{1}{4}A_{max}+\frac{3}{4}A_{average}$$

(10) $$T_{average}=A_{average}$$

**Figure 7:**
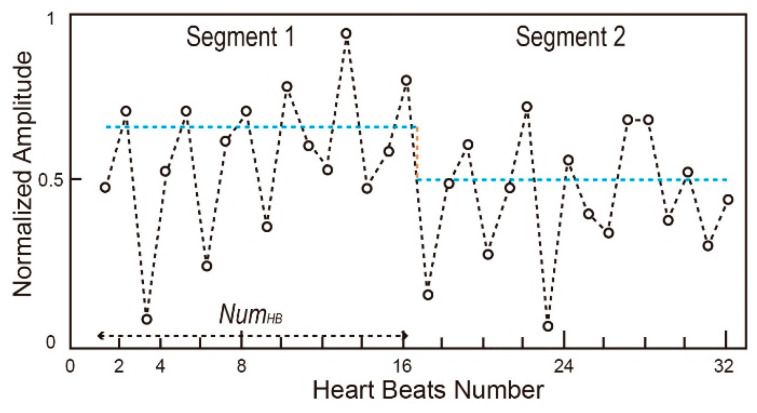
EDR segment and threshold.

Step 3: Count the number of times that the EDR signal crosses upward the segmented threshold lines, as shown in Figure 7. When the respiration number is high (i.e., larger than $Num_{Th.}$), $T_{max}$ is used for the counting. When the respiration number is low (i.e., smaller than $Num_{Th.}$), $T_{average}$ is used for the counting. $Num_{resp.}\;$is the obtained number of breaths.

Step 4: The respiration rate (i.e., bpm) is calculated by averaging $Num_{seg}\;$segments (e.g., 7).

(11) $$Bpm=\frac{\sum_{i=1}^{Num_{seg}}Num_{resp.i}}{Time}\times 60$$

Step 5: The respiration rate is updated by moving to the next $Num_{seg}\;$segments by sliding one segment forward.

The proposed adaptive threshold-based EDR estimation method is based on the counting method and therefore has very low computational complexity compared with the Fourier transform based method. In the meanwhile, it addresses the issue of the conventional counting method and is able to achieve higher estimation accuracy.

## 4. Processor Implementation

**Figure 8:**
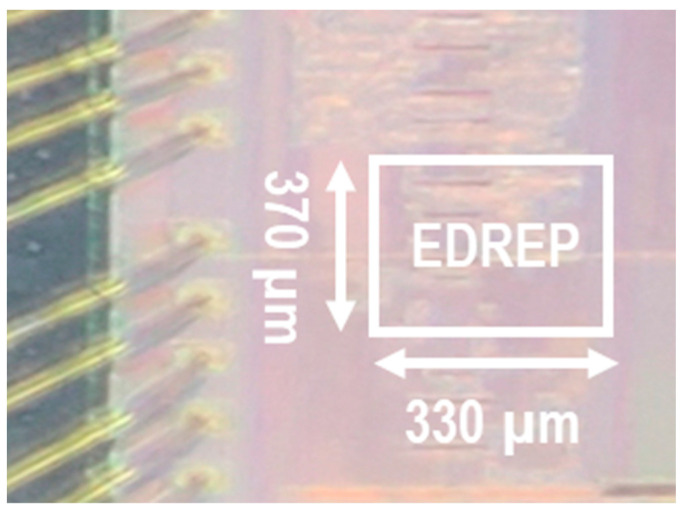
The die photo of the proposed EDR estimation processor (EDREP).

The proposed EDR estimation processor is implemented and fabricated using a 55 nm process technology, and the die photo is shown in Figure 8.

**Figure 9:**
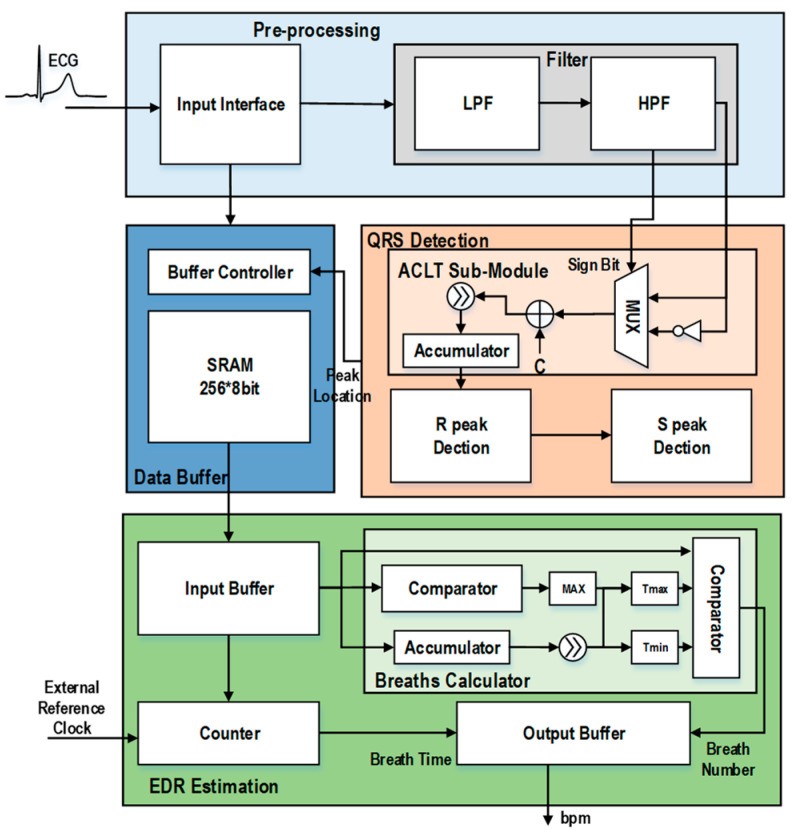
Architecture of the proposed EDR estimation processor.

The hardware architecture of the proposed EDR estimation processor is shown in Figure 9. The overall architecture includes four parts: the pre-processing module, the data buffer module, the QRS detection module and the EDR estimation module.

First, the ECG signal is fed into the processor through the data interface. It goes to two modules: the filter module and the data buffer module. The former is for performing signal de-noising, and the latter is for storing the original ECG signal for later use. After filtering, the signal goes to the QRS detection module to detect the R peak and the S peak. After that, the obtained R peak and S peak locations will be sent to the buffer controller for fetching the corresponding data in the original ECG signal and send it to the EDR estimation module. The EDR estimation module will then calculate the respiration rate using an external reference clock. In the design of the proposed EDR estimation processor, we proposed several design optimizations in the QRS detection module and the EDR estimation module.

### 4.1. Implementation of QRS Detection Module

In the QRS detection module, the signal from the filter is sent to the ACLT sub-module, which is used to enhance the feature of the R peak to facilitate its detection. The R peak detection sub-module detects the R peak using the refractory period refreshing mechanism. After a R peak is detected, the amplitude of R peak and the amplitude of S peak will be determined by checking the original ECG signal stored in the data buffer.

In the implementation of the QRS detection module, the ACLT sub-module involves the computation of the absolute value. This is implemented using inverters and multiplexers, as shown in Figure 9. A sign-bit check block is designed. When the sign bit is ‘0’, the input will be directly output without any processing. When the sign bit is ‘1’, all bits will be inverted. Supposedly, a ‘1’ needs to be added to the result to obtain the absolute value of a negative number. However, we have skipped this operation to save more power consumption, as we found that this does not affect the detection accuracy.

### 4.2. Implementation of EDR Estimation Module

The EDR estimation module includes three sub-modules: input buffer, breaths calculator and output buffer. The input buffer is used to store all the R-S peaks obtained in each segment. When there is a complete segment, the breaths calculator will use the R-S peak in a segment to calculate the threshold and obtain the breath number (as described in Section 3 and using the parameters in Table 2). The EDR estimation module also includes a counter to obtain time information based on an external reference clock to calculate bpm. Using the total number of breaths and the time of segment obtained, the bpm can be calculated.

For the implementation of the EDR estimation module, as the heart rate and respiratory rate of human are usually low (e.g., 1.3 Hz and 0.3 Hz, respectively), there is no need to pursue a high degree of parallelism. Therefore, when designing the EDR estimation module, we reused the comparators and the adders for both threshold calculation and counting, as the threshold calculation needs to calculate the maximum value by comparison and the counting needs to compare the EDR signal with the threshold. This does not affect the real-time operation of the EDR estimation, but saves significant hardware resource.

## 5. Experimental Results

### 5.1. Performance of QRS Detection and EDR Estimation

The CEBS database [28] is used to evaluate the performance of the proposed EDR estimation processor, which is a database commonly used to evaluate the performance of EDR estimation. This database contains ECG signals for EDR and respiration signals generated by the thoracic piezoresistive band for reference. As in another work [10], the data records b001 to b020 in the database are used for verification. These data are recorded when the tester stayed supine and awake. We used the Lead-II data from this dataset for the test. Furthermore, for a fair comparison, we evaluate the proposed method on the MIT-BIH Polysomnographic Database (MIT-BIH slpdb) [29] with the same evaluation method as in [30].

In addition to the CEBS database, we have also used the MIT-BIH Arrhythmia Database to separately evaluate the performance of the proposed QRS detection [31], as the CEBS database does not provide the labelling of the R peaks of the ECG signals. The MIT-BIH Arrhythmia Database contains 48 30-min records. The R peaks are well labeled in this database.

For the evaluation of the QRS detection alone, all the records in the MIT-BIH Arrhythmia Database have been used. The total number of errors is 893, of which FP (i.e., the number of wrongly detected R peaks) is 453 and FN (i.e., the number of undetected R peaks) is 440. As shown in Table 1, compared with the previous results of M1~M3, both FP and FN are reduced, and the total number of errors is reduced from 1900 to 893. The proposed QRS detection achieves an accuracy of 99.18%, which is calculated using (12), where TP means true positive.

(12) $$\mathrm{Accuracy}\left(\mathrm{Acc}\right)=\frac{\mathrm{TP}}{\mathrm{TP}+\mathrm{FP}+\mathrm{FN}}\times 100\%$$

For the evaluation of the proposed EDR estimation processor, as in other work the mean absolute error (MAE) between the bpm estimated by EDR and the reference bpm is used as the metric, which is calculated using (13).

(13) $$\mathrm{MAE}\;=\left|{\mathrm{bpm}}_{\mathrm{reference}}-{\mathrm{bpm}}_{\mathrm{edr}}\right|$$

As shown in Table 3, the obtained MAE is 0.73 on CEBS database and 1.2 on MIT-BIH Polysomnographic Database, respectively. For comparison, we have also used the conventional fixed threshold methods (both maximum value based and average value based) to estimate the respiratory rate. The obtained MAE are 1.62 and 0.83, respectively. This demonstrates the effectiveness and advantage of the proposed adaptive threshold based EDR estimation method. 

We have also compared our design with other EDR estimation methods. As shown in Table 4, the proposed design achieves better MAE than the compared designs.

However, the limitation of this work is that it does not consider the factors such as body position and stress condition, which may affect the performance of the proposed QRS detection method and EDR estimation method. We will investigate the impact of these factors in our future work.

### 5.2. Performance of Proposed Processor

**Figure 10:**
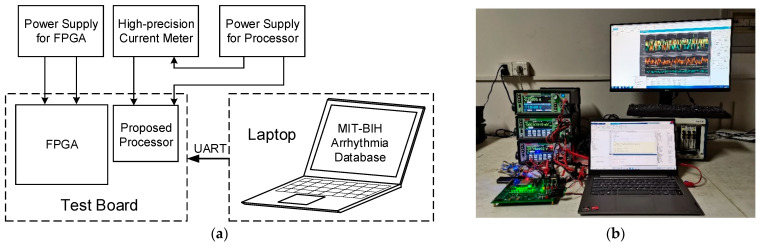
Test setup. (**a**) Block diagram. (**b**) Photo of the environment.

To measure the power consumption of the proposed EDR estimation processor, we run the processor with the data from the MIT-BIH Arrhythmia Database. The test setup is shown in Figure 10. The data is sent from the laptop to the test board through the UART interface. The signals for processor control, e.g., clock, are generated by FPGA on the test board. We measured the operating current of the processor at 1.08 V and 10 kHz, and obtained the average power consumption (which is only 354 nW) by calculating the multiplication result of the supply voltage (1.08 V) and the average current recorded by the high-precision current meter. The proposed design has demonstrated a record-low power consumption and can be integrated in a wearable sensor for ultra-low power and high accuracy respiration monitoring.

## 6. Conclusions

This work proposes a high accuracy and ultra-low power EDR estimation processor. Several techniques have been proposed to improve the accuracy with low computational complexity, including QRS detection using refractory period refreshing and adaptive threshold EDR estimation. The proposed processor has been implemented and fabricated using a 55 nm process technology. It achieves high QRS detection accuracy (99.18%) and low EDR estimation MAE error (0.73 on CEBS database), while demonstrating a record-low power consumption (354 nW) for the respiration monitoring. The proposed processor can be integrated in a wearable sensor for ultra-low power and high accuracy respiration monitoring.

## Figures and Tables

**Table 1:** Result of QRS detection method.

No.	Approach	FP	FN
M1	High threshold with refractory period	**419**	2103
M2	Low threshold without refractory period	6008	516
M3	Low threshold with refractory period	1013	887
M4	Proposed QRS detection method	453	**440**

**Table 2:** Parameters of hardware design.

Parameter	Meaning	Used Value
$Num_{HB}$	The number of R-S peaks in a segment	16
$Num_{Th.}$	Flag value of the adaptive thresholds	4
$Num_{seg}$	The number of total segments	7

**Table 3:** Comparison of present EDR estimation methods.

Method	Database	No. of Subjects	MAE	Platform
EMBC 2017 [30]	MIT-BIH slpdb ^1^	13	2	STM32F4
EMBC 2018 [10]	CEBS	20	1.1	Software
TBME 2020 [32]	In-house	15	3.57%	Software
Information 2021 [33]	CEBS	20	1.5	Software
**Proposed**	**MIT-BIH slpdb**	**13**	**1.2**	**IC**
	**CEBS**	**20**	**0.73 or 3.03% ^2^**	

^1^ MIT-BIH Polysomnographic Database. ^2^ Interquartile range (IQR) of relative error for comparison with [32].

**Table 4:** Comparison with fixed threshold methods.

Threshold	MAE
Maximum value based	1.62
Average value based	0.83
Proposed EDR method	0.73

## Data Availability

MIT-BIH Arrhythmia Database: https://www.physionet.org/content/mitdb/1.0.0/; CEBS Database: https://www.physionet.org/content/cebsdb/1.0.0/; MIT-BIH Polysomnographic Database: https://www.physionet.org/content/slpdb/1.0.0/ (all accessed on 15 June 2022).
